# Retraction: Silencing microRNA-27a inhibits proliferation and invasion of human osteosarcoma cells through the SFRP1-dependent Wnt/β-catenin signaling pathway

**DOI:** 10.1042/BSR20182366_RET

**Published:** 2026-05-18

**Authors:** Yuanyuan Shi, Junfeng Li, Yu Mu, Lina Zhang, Xue Chen, Si Chen

**Affiliations:** 1 Department of Nursing, The Second Hospital of Jilin University, Changchun 130041, P.R. China; 2 Department of Clinical Laboratory, The Second Hospital of Jilin University, Changchun 130041, P.R. China

**Keywords:** Invasion, MicroRNA-27a, Osteosarcoma, Proliferation, SFRP1, Wnt/β-catenin signaling pathway

This article is being retracted from *Bioscience Reports* at the request of the Editor-in-Chief and the Editorial Board.

This follows the receipt of a notification from a reader, alerting the Editorial Board to irregularities in the Western Blot images of Figures 1C, 2C, 3E, 3F, 6C and 7C. Specifically, the Western Blot images are marked by high contrast, an absence of background texture and the irregular appearance of the bands themselves. Upon investigation, the Editorial Office identified additional concerns relating to the unexplained replacement of data and an instance of high similarity between the replaced, unpublished data of Figure 4 with Figure 1A from Li et al. 2017 (DOI: 10.1002/jcb.26375).

The authors have been contacted with regard to the retraction and have not responded to the Journal's queries or the concerns raised. Given the extent of the issues raised, the Editorial Board stands by the decision to retract the article.

